# Self-Healable Electro-Conductive Hydrogels Based on Core-Shell Structured Nanocellulose/Carbon Nanotubes Hybrids for Use as Flexible Supercapacitors

**DOI:** 10.3390/nano10010112

**Published:** 2020-01-06

**Authors:** Huixiang Wang, Subir Kumar Biswas, Sailing Zhu, Ya Lu, Yiying Yue, Jingquan Han, Xinwu Xu, Qinglin Wu, Huining Xiao

**Affiliations:** 1 College of Materials Science and Engineering, Joint International Research Lab of Lignocellulosic Functional Materials, Nanjing Forestry University, Nanjing 210037, China; whx9111@163.com (H.W.); zhusailing@njfu.edu.cn (S.Z.); luyajiangsu@163.com (Y.L.); 2 Laboratory of Active Bio-based Materials, Research Institute for Sustainable Humanosphere, Kyoto University, Uji, Kyoto 611-0011, Japan; subir.biswas.88a@st.kyoto-u.ac.jp; 3 College of Biology and Environment, Nanjing Forestry University, Nanjing 210037, China; yue@njfu.edu.cn; 4 School of Renewable Natural Resources, Louisiana State University, Baton Rouge, LA 70803, USA; wuqing@lsu.edu; 5 Department of Chemical Engineering, University of New Brunswick, Fredericton, NB E3B 5A3, Canada; hxiao@unb.ca

**Keywords:** cellulose nanofibers, carbon nanotube, polyaniline, hydrogels, supercapacitor

## Abstract

Recently, with the development of personal wearable electronic devices, the demand for portable power is miniaturization and flexibility. Electro-conductive hydrogels (ECHs) are considered to have great application prospects in portable energy-storage devices. However, the synergistic properties of self-healability, viscoelasticity, and ideal electrochemistry are key problems. Herein, a novel ECH was synthesized by combining polyvinyl alcohol-borax (PVA) hydrogel matrix and 2,2,6,6-tetramethylpiperidine-1-oxyl (TEMPO)-cellulose nanofibers (TOCNFs), carbon nanotubes (CNTs), and polyaniline (PANI). Among them, CNTs provided excellent electrical conductivity; TOCNFs acted as a dispersant to help CNTs form a stable suspension; PANI enhanced electrochemical performance by forming a “core-shell” structural composite. The freeze-standing composite hydrogel with a hierarchical 3D-network structure possessed the compression stress (~152 kPa) and storage modulus (~18.2 kPa). The composite hydrogel also possessed low density (~1.2 g cm^−3^), high water-content (~95%), excellent flexibility, self-healing capability, electrical conductivity (15.3 S m^−1^), and specific capacitance of 226.8 F g^−1^ at 0.4 A g^−1^. The fabricated solid-state all-in-one supercapacitor device remained capacitance retention (~90%) after 10 cutting/healing cycles and capacitance retention (~85%) after 1000 bending cycles. The novel ECH had potential applications in advanced personalized wearable electronic devices.

## 1. Introduction

Nowadays, with the rapid development of personal wearable electronic devices, miniaturized energy storage devices with mechanical flexibility and even self-healing functions have attracted more and more attention; among them, the supercapacitor is a research hotspot [1,2]. The supercapacitor is a promising new energy storage device, which combines the advantages of high power from double-electric layer capacitors and high energy from batteries [3]. However, traditional supercapacitors are susceptible to various unavoidable mechanical deformations and cannot meet the needs of flexible wearable electronic devices [4]. Therefore, flexibility and self-healing characteristics are requirements for wearable energy storage devices. In order to integrate flexibility, self-healability, and electrochemical performance into a supercapacitor device, searching suitable electrode and electrolyte material has become a key to the manufacture of intelligent devices [5].

Hydrogels are considered as ideal candidates because of their flexible three-dimensional (3D) networks, high deformability, and hydrophilic property [6]. Firstly, hydrogel polymer network with high-water content dissolves ions and provides high ion conductivity, which can be compared with the ionic conductivity of a liquid while maintaining the solid shape and size to avoid liquid leakage during various mechanical deformation. It is an ideal choice for flexible supercapacitor electrolyte and separator materials [7]. Secondly, electrically conductive hydrogel (ECH) is a new functional material that combines flexible 3D hydrogel network with conductive nano-fillers. Due to the inherent porous structure, excellent conductivity, and flexibility of the ECH, it is the perfect choice for flexible electrode materials. The continuous conductive network inside the ECH provides electron transfer pathways. The hierarchical pore structure can ensure sufficient contact between the active material and the electrolyte ions to improve the electrochemical performance [8]. Finally, a supercapacitor assembled from the hydrogel-based electrode and electrolyte, because of the inherent viscoelasticity and flexibility of the hydrogel, will possess an improved interface between the electrode and the electrolyte [9]. However, the related researches on self-healable and flexible hydrogel-based supercapacitors are limited so far [2].

A novel freestanding and moldable hydrogel with excellent self-healing performance was synthesized by incorporating cellulose nanofibers (CNFs) into polyvinyl alcohol-borax (PVA) hydrogel matrix. Conductive materials were incorporated into self-healable PVA hydrogels to form multifunctional ECHs, which had potential applications in flexible and self-healable supercapacitors. In our present study, carbon nanotubes (CNTs) and polyaniline (PANI) were the electrochemically active materials in electrodes. 2,2,6,6-tetramethylpiperidine-1-oxyl (TEMPO)-cellulose nanofibers (TOCNFs) were the bio-dispersant that helped CNTs disperse in water. PANI was coated on the surface of TOCNF-CNT nano-hybrids through in-situ oxidative polymerization to form “core-shell” TOCNF-CNT@PANI composites. These composites built a hierarchically reinforced and conductive 3D network in the self-healable PVA hydrogel matrix. The hydrogel-based electrodes and electrolytes were used to assemble symmetrical solid-state all-in-one supercapacitors with flexible and fast self-healable performances.

## 2. Materials and Methods

### 2.1. Materials

Bleached wood pulp was provided by Nippon Paper Chemicals Co., Ltd. (Tokyo, Japan). 2,2,6,6-tetramethylpiperidine-1-oxyl (TEMPO, C_9_H_18_NO), sodium bromide (NaBr), sodium hypochlorite solution (NaClO, 6–14% active chlorine), ammonium persulfate (APS, H_8_N_2_O_8_S_2_), aniline monomers (ANI, C_6_H_7_N), Sodium hydroxide (NaOH), Potassium hydroxide (KOH), polyvinyl alcohol (PVA, *M*_w_ = 124,000–186,000 g mol^−1^, 99% hydrolyzed), sodium tetraborate decahydrate (Borax, Na_2_B_4_O_7_·10H_2_O, 99.5% purity) were purchased from Aladdin Chemical Reagent Co., Ltd., Shanghai, China. The carbon nanotubes (CNTs) were obtained from Nanotech Port Co., Ltd., Shenzhen, China (the purity >97%; the diameter in the range 10–20 nm; the length in the range 30–100 μm). They were all the analytical grade. Deionized (DI) water was used in all the preparations.

### 2.2. Preparation of TOCNFs, TOCNF-CNT Nanohybrids, and TOCNF-CNT@PANI Nanohybrids

The bleached wood pulp solution was mixed with TEMPO and NaBr by means of mechanical stirring. Then, NaClO (15 mmol g^−1^ cellulose) solution was added to initiate an oxidation reaction. The value of pH should be maintained at 10.5 by adding 1 M NaOH solution in the reaction process. After the oxidation reaction, the achieved fibers were washed by DI water and ultrasonicated at 800 W power for 3 min to obtain TOCNFs [10]. The concentration of final homogenized suspension was adjusted to 0.6 wt%.

A total of 0.5 g CNT powers was added to the 62.5 g 0.6 wt% TOCNFs suspensions at the mass ratios m(CNTs):m(TOCNFs) = 1:0.75 while keeping mechanical agitation for 30 min. Then, they were diluted to 500 mL water and ultrasonicated for 10 min at 400 W power in an ice bath to achieve TOCNF-CNT nanohybrids homogenized aqueous dispersion. The sonication was carried out by a high-energy ultrasonic cell disruptor.

TOCNF-CNT@PANI nanohybrids were synthesized based on TOCNF-CNT nanohybrids as a template and APS as oxidant through in-situ polymerization in an acidic water medium, as follows. First, 50 mL 1 M HCl solution containing 0.875 g ANI (0.0094 mol) was added into the 500 mL TOCNF-CNT nanohybrids dispersions and stirred for 10 min in an ice bath. Next, 50 mL 1 M HCl solution containing 2.68 g APS (0.01175 mol, mole ratio of ANI:APS = 1:1.25) was added slowly to the mixture in 30 min. During the process, the uniform mixture was slowly converted to blue tone, indicating polymerization; then, the solution was kept stirring for 6 h and stand for 12 h in an ice bath to complete polymerization. Finally, the obtained TOCNF-CNT@PANI nanohybrids were washed by vacuum filtration, and the excess unreacted ions were removed. The washed product was transferred to dialysis tubes and dialyzed to pH = 7.

A similar method was used to synthesize TOCNF-CNT@PANI nanohybrids containing different PANI content. The mass ratio of ANI monomers to TOCNF-CNT nanohybrids were 0:1, 1:1, 2:1, and 3:1, and the corresponding TOCNF-CNT@PANI nanohybrids samples were denoted TOCNF-CNT, TOCNF-CNT@PANI-1, TOCNF-CNT@PANI-2, and TOCNF-CNT@PANI-3, respectively.

### 2.3. Preparation of TOCNF-CNT@PANI/PVA Composite Hydrogel

Two grams PVA and 100 mL DI water were stirred for 1 h at 90 °C to achieve a transparent PVA aqueous solution. Then, the solution was added into TOCNF-CNT, TOCNF-CNT@PANI-1, TOCNF-CNT@PANI-2, and TOCNF-CNT@PANI-3 nanohybrids aqueous dispersion and stirred to form homogeneous dispersion, respectively. According to m (PVA):m (borax) = 4:1, borax powders were added slowly into the mixture with stirring at 90 °C until the composite became sticky. The composite showed viscoelastic performance slowly with the decrease of temperature, indicating the formation of the final composite hydrogels. The corresponding hydrogels were named TOCNF-CNT/PVA, TOCNF-CNT@PANI/PVA-1, TOCNF-CNT@PANI/PVA-2, and TOCNF-CNT@PANI/PVA-3, respectively.

### 2.4. Fabrication of the Self-Healing Solid-State Supercapacitor

The supercapacitor device was a symmetric configuration, which was assembled by two pieces of TOCNF-CNT@PANI/PVA-2 (5.0 × 1.5 × 0.1 cm^3^) and one piece of TOCNF/PVA (5.0 × 1.5 × 0.1 cm^3^). They were immersed into 6 M KOH solution for 12 h before assembling. In symmetrical supercapacitors, the electrolyte and separator were TOCNF/PVA hydrogels, which were sandwiched in TOCNF-CNT@PANI/PVA-2 hydrogels using as electrodes and current collectors. The interfaces between electrodes and separators could be completely glued into a single thin separating layer as excellent self-healing character, thereby manufacturing an integrated solid-state supercapacitor. The electrochemical measurements of supercapacitors were performed on an electrochemical workstation (CHI 760E, Shanghai, China).

### 2.5. Characterization

The transmission electron microscope (TEM) was carried out by a transmission electron microscope (JEM-1400, Tokyo, Japan) operating at 120 kV. The concentrations of CNTs, TOCNF-CNT, and TOCNF-CNT@PANI water suspensions were 0.05–0.1 wt%. The absorption spectra of CNTs, TOCNF-CNT, and TOCNF-CNT@PANI suspensions were measured at room temperature using an ultraviolet-visible (UV-vis) spectrophotometer (Tu-1810, Purkinje Co., Beijing, China) at a wavelength of 200–1000 nm. Spectra were collected using water as a reference at a scan speed of 0.5 nm s^−1^. Infrared spectra were performed by Fourier transform infrared (FTIR) spectroscopy (Nicolet iS50, Thermo Fisher Scientific Inc., Madison, WI, USA) with attenuated total reflection (ATR) mode. The wavelength range was from 4000 to 500 cm^−1^ at a resolution of 4 cm^−1^. X-ray diffraction (XRD) spectra were obtained by an X-ray diffractometer (Ultima IV, Rigaku, Japan) at 40 kV and 30 mA, and the angle range was 2*θ* = 5~40° at a scanning rate of 5° min^−1^. The microstructure of composite hydrogel was observed by a JSM-7600F scanning electron microscope (Nippon electronics Co., Ltd., Tokyo, Japan) at a voltage of 15 kV.

Uniaxial compression measurements were carried out through a universal mechanical testing machine (CMT4304, Shenzhen, China) on a cylindrical sample (diameter of 20 mm; the height of 5 mm) at a compression speed of 8 mm min^−1^ at room temperature in air. The values of stress (*σ*) and strain (*ε*) were calculated from the force and deformation of the original size of samples. The compressive elastic modulus (*E*_e_) was calculated from the rake angle ratio of the linear part (*ε* < 20%) of the *σ*-*ε* curve. The specific stress (*σ*_s_) was obtained by dividing the density (*ρ*). The energy absorption (*E*_a_) was the integral area of the part under the *σ*-*ε* curve.

Tensile stress-strain measurements were performed using a universal mechanical testing machine (CMT4304, Shenzhen, China) at a pulling rate of 20 mm min^−1^. The hydrogel samples of initial and healed (20 s) in a size of 30 × 15 × 5 mm^3^ were nipped to the tensile machine during the testing process. All the tensile measurements were repeated for three times. The healing efficiencies (*η*_F_) were calculated from the break stress (*F*_healed_) of the healed hydrogel divided that of the initial one (*F*_original_). The healing efficiencies (*η*_K_) were defined from that the break strain (*K*_healed_) of the healed hydrogel sample divided that of the initial one (*K*_original_). (*η*_F_ = *F*_healed_/*F*_original_ × 100%, *η*_K_ = *K*_healed_/*K*_original_ × 100%)

The dynamic rheological properties, including dynamic frequency scanning, dynamic strain scanning, and continuous step strain, were tested by a Rheometer (HAAKE600, Waltham, MA, USA) with a plate diameter of 40 mm and a gap of 500 μm. The dynamic strain range was 0.01 to 100% with angular frequency (*ω*) of 1 Hz. The linear viscoelastic region (LVR) was decided by storage modulus (*G*′), and the *G*′ was independent of the strain in the LVR. In the following measurement of each sample, 1% strain (*γ*) was selected to maintain the dynamic oscillatory deformation within the LVR. In dynamic frequency scanning measurement, the relationship between the shear storage modulus (*G*′), loss modulus (*G*″), and angular frequency (*ω*) were recorded at *ω* = 0.1–100 rad s^−1^, *γ* = 1%, and 25 °C. The complex modulus (G*) was calculated by Equation (1).

(1) $$G=\sqrt{G^{\prime }{}^{2}+G^{\prime \prime }{}^{2}}$$

Continuous step strain tests were conducted to study the recovery property of the hydrogels under the applied shear stress. A procedure to the program was as follows: 1% (800 s) →80% (800 s) → 1% (800 s) → 80% (800 s) → 1% (800 s), the *G*′ and *G*″ versus time were measured at *ω* = 1 Hz and 25 °C.

Conductivity tests of hydrogel electrodes. A square-shaped hydrogel with a size of 1 × 1 × 10 cm^3^ was sandwiched by two pieces of platinum electrodes. The resistance (*R*) values of the hydrogel were decided by current-voltage (*I*–*V*) measurement using an electrochemical workstation (CHI 760E, Shanghai, China). The conductivity (*σ*) was achieved from Equation (2):

(2) $$\sigma =\frac{1\times d}{R\times S}$$

where *σ* was the conductivity (S m^−1^), *R* was the resistance (Ω), *d* was the length (m), and *S* was the cross-sectional area (m^2^) of the sample, respectively.

Electrochemical measurements of the hydrogel-based electrodes were performed on a three-electrode system using an electrochemical workstation (CHI 760E, Shanghai, China). The working electrode, the counter electrode, the reference electrode, and electrolyte were the TOCNF-CNT@PANI/PVA-2 hydrogel (1.5 g), platinum plate electrode, mercury/mercury oxide (Hg/HgO) electrode, and 6 M KOH aqueous solution, respectively. The cyclic voltammetry (CV) test was carried out at scan rates of 40 mV s^−1^ from −0.2–0.8 V, the galvanostatic charge-discharge (G-CD) test was performed over the voltage range of −0.2–0.8 V at a current density of 0.4 A g^−1^, and electrochemical impedance spectra (EIS) test was measured over the frequency range from 0.01 Hz to 100 kHz at open circuit potential (alternating current perturbation voltage was 5 mV). The specific capacitance (*C*_s_) values were calculated from the G-CD curves using Equation (3):

(3) $$C_{s}=\frac{I\Delta t}{m\Delta V}$$

where *C*_s_ represented the specific capacitance (F g^−1^), *I* represented the discharge current (A), Δ*t* represented the time of discharge (s), Δ*V* represented the voltage of discharge (V), and *m* represented the mass of active materials (g).

## 3. Results and Discussion

### 3.1. Synthesis Process and Mechanism of TOCNF-CNT@PANI/PVA Composite Hydrogels

**Figure 1:**
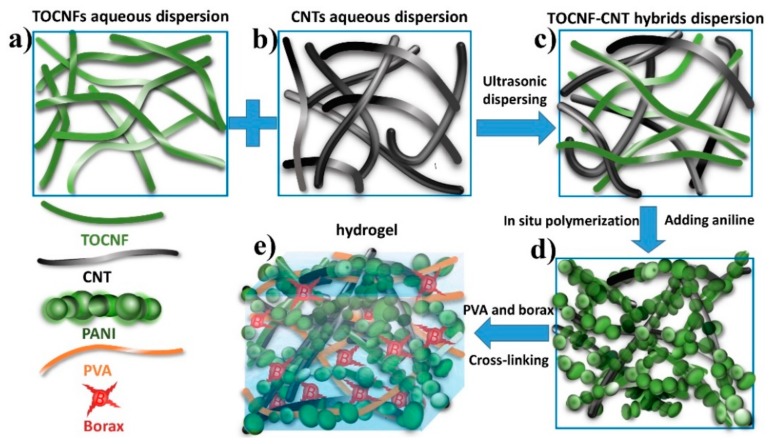
Schematic of the fabrication process of TOCNF-CNT@PANI/PVA composite hydrogel. (**a**) TOCNFs aqueous dispersion, (**b**) CNTs aqueous dispersion, (**c**) TOCNF-CNT nanohybrids aqueous dispersion, (**d**) TOCNF-CNT@PANI nanohybrids aqueous dispersion, (**e**) TOCNF-CNT@PANI/PVA composite hydrogel.

The preparation process of hierarchical 3D network TOCNF-CNT@PANI/PVA composite hydrogel is illustrated in Figure 1. Firstly, TOCNF suspensions were prepared through a TEMPO oxidation treatment in an aqueous system following an ultrasonication treatment. TEMPO oxidizes the hydroxymethyl group at the glycose C_6_ position in the cellulose chain to a more active carboxyl group [11]. Secondly, the homogeneous TOCNF-CNT nanohybrid dispersions were obtained by mixing CNT powders with TOCNF dispersions through ultrasonication. Not only the fluctuation of counter ions on the surface of TOCNF fibers induced the dipoles of the carbon lattice in CNTs, but also the carboxyl groups of TOCNFs produced electrostatic repulsion, which ensured the stabilization of CNTs in water [10]. Thirdly, TOCNF-CNT with excellent dispersibility and high specific surface area were used as nanocarrier of PANI. TOCNF-CNT@PANI nanohybrids were synthesized by in-situ chemical polymerization with APS as oxidant and TOCNF-CNT as a biological template in acid medium. PANI formed a wholly uniform coating layer around TOCNF-CNT nanohybrid bundles (Figure 1d), which would bring enough pseudo-capacitance [12]. Finally, PVA and borax were introduced into the TOCNF-CNT@PANI nanohybrid dispersions to achieve TOCNF-CNT@PANI/PVA composite hydrogel through cross-linking reaction. Borax would decompose into B(OH)_4_^−^ ions in water, and B(OH)_4_^−^ ions would create a reversible connection between the CNF-CNT@PANI composite fibers and the PVA molecular chains, forming a dynamic 3D network structure in the hydrogel [6]. The hierarchical 3D network inside hydrogel is illustrated in Figure 1e; the CNTs provided the fast electron transport path, and the nano-coating layer of PANI-ensured electrons could only pass through a very short distance to the CNT networks with high conductivity, which improved the electrochemistry of TOCNF-CNT@PANI nanohybrid. In addition, the borate ions could combine with the adjacent hydroxyl groups to form dynamic cross-linking between PVA chains and TOCNF-CNT@PANI nanohybrids. The dynamic PVA-borate cross-linking network provided hydrogel with the moldable and self-healing performance. The TOCNF-CNT networks provided an additional platform to improve strength, toughness, and conductivity. The chain entanglement and hydrogen bonding between TOCNF-CNT@PANI nanohybrids and PVA formed a hierarchical 3D network in TOCNF-CNT@PANI/PVA hydrogels.

### 3.2. Dispersion State and Chemical Analysis of TOCNF-CNT and TOCNF-CNT@PANI Nanohybrids

**Figure 2:**
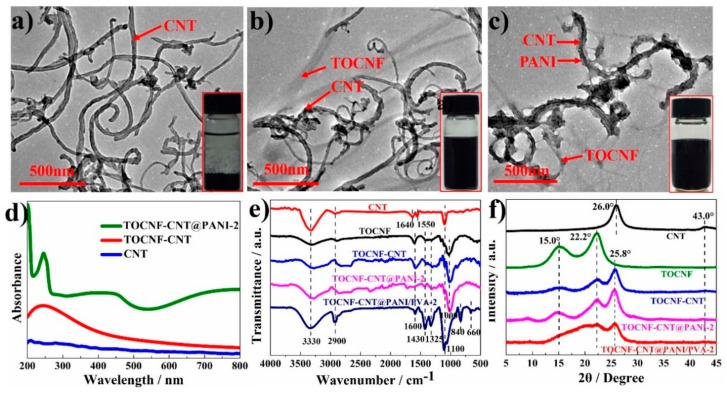
Transmission electron microscope (TEM) images of (**a**) CNTs, (**b**) TOCNF-CNT nanohybrids, (**c**) TOCNF-CNT@PANI nanohybrids, and the inset in each image represents the corresponding dispersion state of each suspension at the concentration of 0.5 wt%; (**d**) Ultraviolet-visible (UV-vis) spectra of neat CNTs, TOCNF-CNT, and TOCNF-CNT@PANI-2 nanohybrids in water. (**e**) Fourier transform infrared (FTIR) spectra and (**f**) X-ray diffraction (XRD) patterns of TOCNFs, CNTs, TOCNF-CNT, TOCNF-CNT@PANI-2 nanohybrids, and TOCNF-CNT@PANI/PVA-2 composite hydrogel.

The microstructure morphologies of CNTs, TOCNF-CNT, and TOCNF-CNT@PANI-2 nanohybrids are shown in Figure 2a–c. Due to the hydrophobic surface and strong van der Waals forces between CNT fibers, it was difficult for the pristine CNTs to disperse well in water [13]. It was clear to see that pristine CNTs formed densely agglomeration and precipitation in water (Figure 2a and insert). After introducing TOCNFs into CNT suspension, as shown in Figure 2b, the CNTs (length: 500–700 nm, diameter: 20–30 nm) dispersed in an individual form without aggregation in water with the help of TOCNFs (length: 1 μm, diameter: 20–30 nm).

Figure 2c shows nodular-structure. PANI particles were deposited on the surface of TOCNF-CNT nanohybrids, forming a continuous shell structure with a high aspect ratio. The in-situ deposited aniline fiber could grow a fresh polymer, then initiate the continuous growing process and form a block precipitate, and the initial seed morphology would be transcribed on a long scale [14]. TOCNF-CNT@PANI composite fiber with a “core-shell” structure was the prototype of the 3D hierarchical conductive network. The PANI grew along the TOCNF-CNT templates to form the “shell”, which was carried by TOCNF-CNT fibers, and dispersed in water to construct a hierarchical 3D conductive network. It was noted that the continuous conductive networks fabricated by TOCNF-CNT@PANI composite fibers were obligatory to synthesize the ECHs with high conductivity and enhanced mechanical performances [15].

The UV-vis spectroscopy of CNT, TOCNF-CNT, and TOCNF-CNT@PANI-2 complexes are shown in Figure 2d. Due to the poor dispersibility and high aggregation, the absorption peak of pure CNT suspension was much weaker than that of the TOCNF-CNT nanohybrids at the same CNT concentrations (0.015 wt%), suggesting that TOCNFs improved significantly the dispersibility of CNTs in an aqueous medium. The absorption peak of TOCNF-CNT nanohybrids was around 262 nm in the UV-vis spectrum, which could be assigned to the π−π transitions of CNTs and showed a more uniform dispersion state of CNTs [16]. Two peaks of TOCNF-CNT@PANI-2 at 285 and 475 nm were from the transition of π−π inter-band and the polaron band of PANI, respectively. The end band (~800 nm) of the TOCNF-CNT@PANI-2 complexes was smooth because of the cross-linking of polymer chains, indicating that it was successful in doping PANI into the conductive state [17].

The FTIR spectra of TOCNFs, CNTs, TOCNF-CNT nanohybrids, TOCNF-CNT@PANI-2 composite, and TOCNF-CNT@PANI/PVA-2 hydrogel is shown in Figure 2e. For all the samples, the broad bands at around 3330 cm^−1^ and sharp bands at around 2900 cm^−1^ were generally due to O-H stretching of the hydrogen bonds and asymmetrically stretching vibration of C–H in the CH_2_ group, respectively [18,19,20,21]. The peaks around 1000 cm^−1^ and 1600 cm^−1^ were ascribed to the C–O–C vibration and the carbonyl functional groups of cellulose [22,23,24]. For the spectra of neat CNTs, the characteristic absorption around 1640 cm^−1^ was assigned to the quaking of the carbon skeleton [25,26]. For the spectra of TOCNFs, the peaks at 1430 cm^−1^ and 1325 cm^−1^ were assigned to the hydrogen bonding and CH_2_ wagging [27]. The typical absorption peak of TOCNF-CNT-2 nanohybrids was analogous to that of TOCNFs, which was due to the absorption peak of TOCNFs covering the absorption peak of the CNTs in the FTIR spectra, revealing the CNTs were successfully combined with TOCNF bio-templates [28]. Comparing with the spectra of pure CNTs, the C–O–C bending band shifted from 1100 cm^−1^ to 1000 cm^−1^ (TOCNF-CNT-2 nanohybrids), confirming the existence of hydrogen bonding between TOCNFs and CNTs [29].

After in situ polymerization, the peaks at 1430 and 1325 cm^−1^ were assigned to the C = C vibration deformation of the quinoid ring and benzenoid ring, respectively [30]. For TOCNF-CNT@PANI-2 complexes, the N−H bonding vibration caused the major peaks at 3330 cm^−1^ shifted to lower wavenumbers, indicating that the TOCNF-CNT nanohybrids were coated with PANI [31]. Further, the vanishing of the sharp band at 1600 cm^−1^ from the carbonyl functional groups of cellulose revealed that PANI coated onto TOCNF-CNT nanohybrids successfully. For TOCNF-CNT@PANI/PVA-2, the distinct peaks at 1430 and 1325 cm^−1^ were attributed to the asymmetric B−O−C bonding, 840 cm^−1^ was assigned as B–O bonding of free B(OH)_4_^−^, and 660 cm^−1^ was ascribed to B–O–B bonding in the borate molecule networks, suggesting the presence of borax and borate [32], which further confirmed the existence of borate cross-linking network between the PVA molecule chains, TOCNF−CNT@PANI nanohybrids, and borate within the hydrogels [33].

Figure 2f shows the XRD diffraction patterns of CNTs, TOCNFs, TOCNF-CNT nanohybrids, TOCNF-CNT@PANI-2 nanocomposite, and TOCNF-CNT/PVA-2 hydrogel samples. A diffraction peak of CNTs at 2*θ* = 26° arose from interlayer spacing (002), reflecting the characteristic of graphite. The diffraction peak at 2*θ* = 43° arose from in-plane crystal lattice (100) [34]. In the XRD diffraction patterns of TOCNFs, a sharp peak and a broad peak at 2*θ* = 22.2° and 15.0°, attributed to (002) and (101) planes, suggested the crystallization from cellulose I [22]. Comparing with the XRD profile of pure CNTs, the TOCNF-CNT nanohybrids showed two additional peaks at 2*θ* = 15.0° and 22.2°, reflecting the characteristic of cellulose I. These observations suggested that CNTs and TOCNFs were combined and remained integrality [35].

For the XRD spectra of TOCNF-CNT@PANI-2 nanocomposite, due to in situ polymerization of ANI monomers, the peaks located at around 2*θ* = 15.0° and 22.2° were wider than that of TOCNF-CNT nanohybrids, which could be attributed to the overlapping of diffraction peaks at 2*θ* = 19.4° and 14.9° from (020) and (011) crystal planes of PANI [36]. The peak intensity at 2*θ* = 25.8° was enhanced, which was due to the overlapping of diffraction peaks of π-π stacking corresponding to the co-facially stacked conjugated backbones from the polymer chains of PANI [37]. After the incorporation of PVA hydrogel, the peaks of CNFs at 2*θ* = 15° disappeared, and a broad new peak emerged at 2*θ* = 22.2°, which contained the diffraction peaks at 2*θ* = 19.4° corresponding to the orthogonal lattice from PVA with semi-crystalline structure. All these revealed the strong interactions between PVA, borax, and TOCNF-CNT@PANI-2 nanocomposite and built a 3D network in the composite hydrogels [38].

### 3.3. Compression Test and Microstructures of Hydrogels

**Figure 3:**
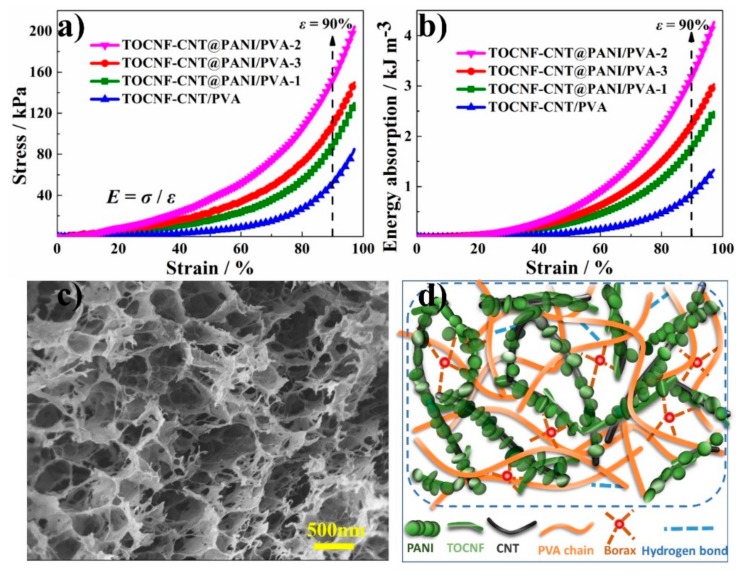
(**a**) Stress-stain curves under compression; (**b**) energy absorption-strain curves of hydrogels; (**c**) SEM image of TOCNF-CNT@PANI/PVA-2 composite hydrogel; (**d**) idealized 3D cross-linking network of TOCNF-CNT@PANI/PVA-2 composite hydrogel.

Figure 3a shows the stress-strain curves of these hydrogels under compression. The measured stresses at the 90% strain level were 52.3 ± 0.3, 86.1 ± 3.9, 108.4 ± 4.3, and 152.3 ± 5.1 kPa for TOCNF-CNT/PVA, TOCNF-CNT@PANI/PVA-1, TOCNF-CNT@PANI/PVA-3, and TOCNF-CNT@PANI/PVA-2, respectively. Thus, the stress of TOCNF-CNT@PANI/PVA-1 hydrogel with PANI at the 90% strain level was almost 1.6-fold than that of TOCNF-CNT/PVA. PANI nanoparticles combined with TOCNF-CNT nanofiber to form TOCNF-CNT@PANI composite fibers with a “core-shell” structure. The composite fibers based on good dispersibility and interfacial adhesion inside the hydrogel effectively transferred the load, thereby improving the mechanical strength of PVA hydrogel [39]. With the increase of PANI content, the stress of TOCNF-CNT@PANI/PVA increased first and then decreased. The stress of TOCNF-CNT@PANI/PVA-3 was 108.4 ± 4.3 kPa, which was lower than that of TOCNF-CNT@PANI/PVA-2 with 152.3 ± 5.1 kPa. This phenomenon could be attributed to the TOCNF-CNT biological template being insufficient to carry and disperse these excess PANI. Aggregated PANI prevented effective cross-linking between PVA and borax and disrupted the integrity of the network in the hydrogel. Under external force, the stress concentration caused by agglomeration would weaken the mechanical strength [40].

The TOCNF-CNT@PANI/PVA-2 possessed the highest mechanical strength in all the hydrogels. Its *σ* value (152.3 ± 5.1 kPa) at *ε* = 90% and *E*_e_ value (61.0 ± 0.8 kPa) in the *σ*-*ε* curve were 2.9-fold and 4.2-fold more than those (*σ* = 52.3 ± 0.3 kPa, *E*_e_ = 14.4 ± 0.3 kPa) of TOCNF-CNT/PVA hydrogel. The specific compressive stress (*σ*_s_) value of TOCNF-CNT@PANI/PVA-2 was 128 kPa cm^3^ g^−1^, which was 2.8-fold larger than that of TOCNF-CNT/PVA with 45.9 kPa cm^3^ g^−1^. In Figure 3b, TOCNF-CNT@PANI/PVA-2 had the largest energy absorption (*E*_a_) value. In the *E*_a_*-ε* curves of hydrogels, the *E*_a_ with *ε* = 90% was selected to compare the mechanical properties of hydrogels. In particular, the *E*_a_ value of TOCNF-CNT@PANI/PVA-2 at *ε* = 90% was 3.2 ± 0.5 kJ m^−3^, which was approximately 4 times larger than TOCNF-CNT/PVA with 0.8 ± 0.4 kJ m^−3^. All the values of strength and physical properties are collected in Table 1.

The improvement of mechanical properties was due to the effective enhancement of TOCNF-CNT@PANI composite fibers with a “core-shell” structure. In addition, the CNTs were entangled with each other to form a lot of contact junctions in the interstitial space between the PVA molecular chain. These contact junctions built the continuous conductive network in the hydrogel matrix. Figure 3c shows the microstructure of TOCNF-CNT@PANI/PVA-2 composite hydrogel, and Figure 3d presents the schematic diagram of the 3D network structure. The composite hydrogel possessed an interconnected porous structure, and each pore had a diameter of 200–500 nm. The wall of the pore was formed by a hydrogel matrix with a thickness of 10–30 nm. The entangled TOCNF-CNT@PANI composite fibers penetrated through the hole wall and built a hierarchical network. The specific framework could effectively promote the transport of electrons, improving the electrical conductivity of the hydrogel. Among them, TOCNFs were important to promote the formation of hierarchical microstructure, which profited from their excellent natural characteristics of hydrophilicity, high aspect-ratio, mechanical strength, and flexibility. TOCNFs combined with CNTs through hydrogen bonding and chain entanglement and served as nanocarriers to disperse CNTs in aqueous media [13]. The well-dispersed TOCNF-CNT nanohybrids were coated by PANI to form TOCNF-CNT@PANI composite fibers with “core-shell” structure. The composite fibers could further improve the mechanical strength and electrical properties of hydrogel [41]. The CNTs in composite fiber could effectively transfer the force from the PVA molecule chains. Moreover, an efficient and stable CNTs electric network could improve the electrical conductivity of TOCNF-CNT@PANI/PVA composite hydrogel. The results showed that the hierarchical network microstructure inside the composite hydrogel increased the interface between the electrolyte and the electroactive material, demonstrating a broad application prospect in flexible electrodes.

### 3.4. Dynamic Viscoelastic Performance of Hydrogels

**Figure 4:**
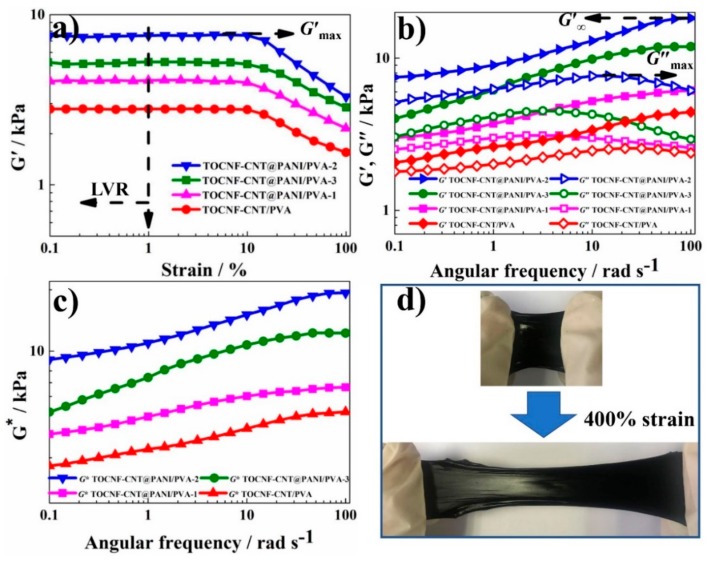
Dynamic viscoelastic properties of hydrogels at 25 °C. (**a**) storage modulus (*G′*) curves based on strain (*γ*) at angular frequency (*ω*) = 1 Hz; (**b**) *G′* and loss modulus (*G′*′) curves based on *ω* at *γ* = 1%; (**c**) complex modulus (G*) curves based on *ω* at *γ* = 1%; (**d**) stretching demonstration of TOCNF-CNT@PANI/PVA-2 hydrogel.

Figure 4a shows the *G*′ curves of hydrogel samples based on strain at *ω* = 1 Hz. Within the LVR, the *G*″ and *G*′ of the hydrogel were independent of strain, as determined by dynamic strain scanning tests. The critical strain (*γ*_c_) of hydrogel was a strain point, where the *G*′ value decreased from the platform value by 5%, indicating deviation from LVR [38]. The *G′* value corresponding to the strain higher than *γ*_c_ would gradually decrease, indicating that the quasi-solid hydrogel had changed to a quasi-liquid state. The *γ*_c_ values of TOCNF-CNT/PVA, TOCNF-CNT@PANI/PVA-1, TOCNF-CNT@PANI/PVA-2, and TOCNF-CNT@PANI/PVA-2 were 2.5%, 2.1%, 1.2%, and 1.5%, respectively. Therefore, in the following dynamic oscillation measurement, the *γ*_c_ value was selected as *γ* = 1%, which could ensure that deformations of the hydrogel samples were within the LVR. For all the hydrogel samples in the LVR, the *G′* values were independent of strain, and the corresponding *G*′_max_ was 2.8, 4.1, 7.5, and 5.1 kPa, respectively (Figure 4a). The *G*′_max_ of TOCNF-CNT@PANI/PVA-1 was 1.5 times that of TOCNF-CNT/PVA. TOCNF-CNT@PANI/PVA-2 possessed the largest *G*′_max_ (7.5 kPa), which was nearly 1.8-fold larger than TOCNF-CNT@PANI/PVA-1 (4.1 kPa) and 1.5-fold greater than TOCNF-CNT@PANI/PVA-3 (5.1 kPa). Incorporation of an appropriate amount of PANI could improve remarkably the stiffness of hydrogel. The shorter the LVR, the closer the sample was to the solid-state. Compared with TOCNF-CNT@PANI/PVA-1 and TOCNF-CNT@PANI/PVA-3, it could be known that the TOCNF-CNT@PANI/PVA-2 possessed a higher *G*′_max_ and shorter LVR, indicating that TOCNF-CNT@PANI/PVA-2 was the strongest hydrogel. The result was consistent with the mechanical strength test.

In order to study the effect of TOCNF-CNT@PANI composite fiber on the viscoelasticity of hydrogels, the *G*′ (elasticity) and *G*″ (viscosity) of hydrogels versus *ω* at *γ* = 1% in the LVR are shown in Figure 4b. As shown, the *G*′ and *G*″ curves of all the hydrogels followed similar trends. With the increase of *ω*, the *G*′ increased monotonically and arrived at plateau value (*G*′_∞_), indicating the formation of neighboring polymer chains entanglements; the *G*″ increased preliminarily to reach the maximum value (*G*″_max_), then decreased gradually. For all the hydrogels, the *G′* values were always higher than the *G*″ values throughout the *ω* range, suggesting hydrogels showed typical solid-like characteristics, indicating that a dynamic cross-linked network was established inside hydrogel [6,42]. The *G*′_∞_ and *G*″_max_ values of TOCNF-CNT/PVA were 4.3 and 2.6 kPa, respectively. After the introduction of PANI, the *G*′_∞_ (6.1 kPa) and *G*″_max_ (3.1 kPa) values of TOCNF-CNT@PANI/PVA-1 were 1.4 and 1.2 times those of TOCNF-CNT/PVA, respectively. It was shown that the combination of PANI and TOCNF-CNT to form a TOCNF-CNT@PANI composite fiber with a “core-shell” structure could significantly improve the viscoelasticity of hydrogel. Comparing between TOCNF-CNT@PANI/PVA-1, TOCNF-CNT@PANI/PVA-2, and TOCNF-CNT@PANI/PVA-3, the TOCNF-CNT@PANI/PVA-2 showed the highest *G*′_∞_ (18.2 kPa) and *G*″_max_ (7.6 kPa). These data of dynamic viscoelastic properties are summarized in Table 2. It showed that an appropriate proportion of PANI could develop a hierarchical network structure and increase viscoelasticity together with TOCNF-CNT. However, excessive PANI would form aggregation due to insufficient TOCNF-CNT to disperse and load. A large amount of PANI blocked the cross-linking between PVA and borax, reducing the dynamic viscoelasticity of the hydrogel [33]. Figure 4c shows the curves of complex modulus (G*) versus *ω*, which provided a clear contrast of viscoelasticity. The trend was TOCNF-CNT@PANI/PVA-2 > TOCNF-CNT@PANI/PVA-3 > TOCNF-CNT@PANI/PVA-1 > TOCNF-CNT/PVA within the entire range of *ω*. The TOCNF-CNT@PANI/PVA-2 showed the highest G*, further proving that TOCNF-CNT@PANI/PVA-2 was the most quasi-solid hydrogel among these hydrogels. In Figure 4d, a piece of rubbery TOCNF-CNT@PANI/PVA-2 hydrogel could be stretched to 400% strain without damage, exhibiting excellent flexibility, viscoelasticity, and efficient energy dissipation capability. Inside hydrogel, flexible PVA chains and long TOCNF-CNT@PANI composite fiber were physically entangled or hydrogen-bonded to build a 3D network, which could unravel and reconstruct the energy dissipating ability of the hydrogel.

### 3.5. Self-Healing Performance of the Hydrogels

**Figure 5:**
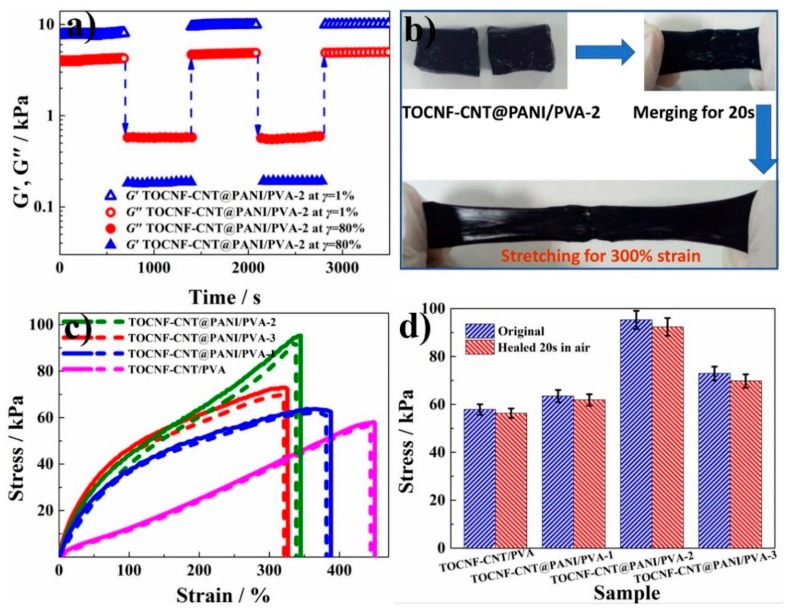
(**a**) The storage modulus (*G′*) and loss modulus (*G″*) dependence of time in continuous step strain measurements. (**b**) Illustration of self-healing property for TOCNF-CNT@PANI/PVA-2 hydrogel. (**c**) Hydrogel stretching curve before and after 20 s self-healing. The solid lines represent the original hydrogels, and the dashed lines represent the self-healed hydrogels. (**d**) Histogram of tensile stress of the original hydrogels and the self-healed hydrogels after healing in the air for 20 s.

The composite hydrogel possessed a dynamic self-healing PVA-borate network, and the TOCNF-CNT@PANI composite fibers network provided an additional platform to strengthen the structure [33]. In Figure 5a, the *G*′ and *G*″ of TOCNF-CNT@PANI/PVA-2 were 8.1 and 4.2 kPa at *γ* = 1%, respectively. The value of *G*′ was larger than that of *G*″, indicating that the elastic character of hydrogel became the dominant factor. When the strain increased to *γ* = 80%, the corresponding *G*′ and *G*″ of TOCNF-CNT@PANI/PVA-2 were 0.2 and 0.6 kPa, respectively. The value of *G*′ was less than that of *G*″, indicating hydrogel turned to the quasi-liquid state. Interestingly, when the strain dropped to 1% again, the corresponding *G*′ and *G*″ immediately restored the original values, indicating that the hydrogel recovered the quasi-solid state. The rapid and repeatable phase transition between the quasi-solid state and quasi-liquid state demonstrated the intrinsic preeminent self-healing capability of the hydrogel. To visualize the self-healing property of the hydrogel, two blocks of TOCNF-CNT@PANI/PVA-2 were pushed together for 20 s, and their contact surfaces would fuse. In addition, after the self-healed hydrogel was stretched to 300% strain, there was no crack at the healing interface (Figure 5b).

The stress-strain curves of hydrogels are shown in Figure 5c. The maximum break strain values of original TOCNF-CNT/PVA, TOCNF-CNT@PANI/PVA-1, TOCNF-CNT@PANI/PVA-2, and TOCNF-CNT@PANI/PVA-3 were 450.5 ± 23.2%, 387.8 ± 19.0%, 345.1 ± 15.1%, and 326.7 ± 12.5%, respectively. The highest tensile stress values of original TOCNF-CNT/PVA, TOCNF-CNT@PANI/PVA-1, TOCNF-CNT@PANI/PVA-2, and TOCNF-CNT@PANI/PVA-3 were 57.9 ± 2.1, 63.5 ± 2.5, 95.3 ± 3.2, and 72.9 ± 2.9 kPa, respectively. The TOCNF-CNT@PANI/PVA-2 composite hydrogel possessed the highest tensile stress, which was 1.6 times that of TOCNF-CNT/PVA hydrogel without PANI, indicating that the proper incorporation of PANI could effectively improve the tensile stress of composite hydrogels. This was consistent with previous measurements of mechanical strength and dynamic viscoelasticity.

To calculate the self-healing efficiency of hydrogels, these self-healed hydrogels after 20 s healing in the air were measured by a tensile test. The tensile curves of the self-healed hydrogels were basically the same as that of the original hydrogels. The maximum break strain values of healed TOCNF-CNT/PVA, TOCNF-CNT@PANI/PVA-1, TOCNF-CNT@PANI/PVA-2, and TOCNF-CNT@PANI/PVA-3 were 443.8 ± 22.1%, 381.1 ± 18.0%, 338.5 ± 13.3%, and 321.0 ± 10.2%, respectively. The corresponding *η*_k_ values were 98.5%, 98.3%, 98.1%, and 98.2%, respectively. The maximum tensile stress of healed TOCNF-CNT/PVA, TOCNF-CNT@PANI/PVA-1, TOCNF-CNT@PANI/PVA-2, and TOCNF-CNT@PANI/PVA-3 were 56.3 ± 2.0, 61.9 ± 2.4, 92.3 ± 3.7, and 69.7 ± 2.8 kPa, respectively. The corresponding *η*_F_ values were 97.2%, 97.5%, 96.8%, and 96.7%, respectively. These data of dynamic viscoelasticity properties are summarized in Table 3, which demonstrated the outstanding self-healing property of as-prepared hydrogels with the dynamic borate-assisted cross-linking network. The complexation between borate and hydroxyl was extremely fast (0.33 s), and TOCNFs and PVA chains contained large number of hydroxyl groups so that the hydrogels could recover quickly [43].

### 3.6. Conductivity Analysis of Hydrogels

**Figure 6:**
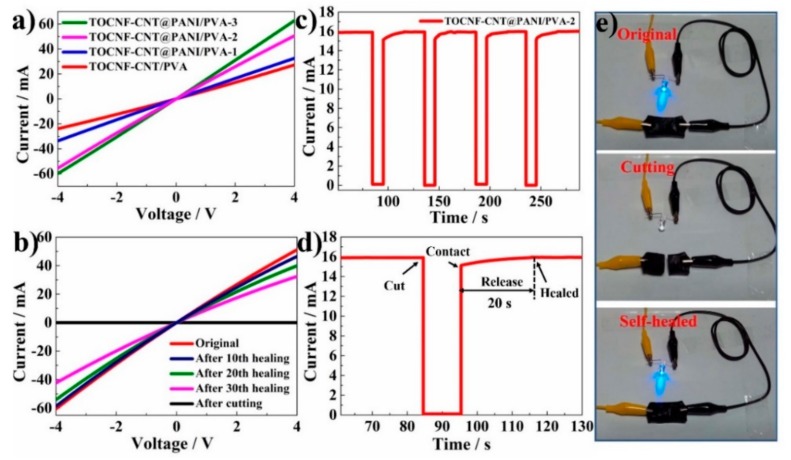
(**a**) Current-voltage (*I–V*) curves of TOCNF-CNT@PANI/PVA with different doping concentrations of PANI. (**b**) *I–V* curves of TOCNF-CNT@PANI/PVA-2 hydrogel after multiple cutting/healing cycles. (**c**) Cycling of the cutting/healing processes for TOCNF-CNT@PANI/PVA-2 at the same location under ambient conditions. (**d**) Time dependence of the electrical healing process by *I-V* measurements under ambient conditions. (**e**) Optical images of TOCNF-CNT@PANI/PVA-2 hydrogel under a cutting/healing cycle in a circuit with a light-emitting diode (LED) bulb.

The composite hydrogel not only possessed excellent mechanical properties and self-healing ability but also possessed outstanding electrical conductivity due to the existence of CNTs and PANI in the hydrogels. The conductivity was quantitatively characterized by the *I–V* measurement at potential ranging from −4 to 4 V. In Figure 6a, the *I–V* curves of composite hydrogels are all linear and non-hysteretic, indicating the excellent electro-conductive character. The conductivity of the TOCNF-CNT@PANI/PVA-3, TOCNF-CNT@PANI/PVA-2, TOCNF-CNT@PANI/PVA-1, and TOCNF-CNT/PVA composite hydrogels were 15.3, 12.8, 8.2, and 6.4 S m^−1^, respectively. The value was superior to phytic acid cross-linked polyaniline/poly(N-isopropylacrylamide) (PANI/PNIPAM) conductive hydrogels (~0.8 S m^−1^) [15]. polyaniline-poly(styrene sulfonate) (PANI-PSS) hydrogels (~10^−2^ S m^−1^) strengthened by sorbitol derivatives (DBS) supramolecular nanofibers [40]. Theoretically, CNTs and PANI were the main active material of conductive network within the composite hydrogels. With PANI as the shell and CNTs as the core, a composite fiber with a “core-shell” structure was formed. The electrical conductivity of the composite fiber was higher than that of bare CNTs fibers [44]. The conductivity of hydrogel increased rapidly when the mass ratio of ANI to TOCNF-CNTs increased from 1:1 to 2:1. However, the conductivity of hydrogel increased slowly, when the mass ratio of ANI to TOCNF-CNTs changed from 2:1 to 3:1. It could be concluded that a 2:1 ratio of ANI and TOCNF-CNTs could form the most perfect conductive network. As the ratio of ANI to TOCNF-CNT increased to 3:1, the TOCNF-CNT skeleton framework was insufficient to load excess PANI, which resulted in a slow increase in conductivity. Consequently, the well-integrating and stability of the TOCNF-CNT@PANI conductive network with the “core-shell” structure offered an effective electron-transfer pathway in the hydrogel. TOCNF-CNT@PANI/PVA-2 was selected for the next experiment based on the previous mechanical test results.

The electrical conductivity’s self-healing efficiency of composite hydrogels was further investigated. The conductivity of original, cutting, and self-healed TOCNF-CNT@PANI/PVA-2 hydrogel was characterized by the *I–V* measurement in Figure 6b. After 10th, 20th, and 30th self-healing, the conductivity of TOCNF-CNT@PANI/PVA-2 hydrogel was 12.8, 11.6, 10.0, and 8.0 S m^−1^, respectively. The self-healing efficiency was calculated by *σ*_r_/*σ*_i_ (*σ*_r_ is the healing conductivity, and *σ*_i_ is the original conductivity) [45]. After 10th, 20th, and 30th self-healing, the self-healing efficiency of TOCNF-CNT@PANI/PVA-2 hydrogel was 90.6%, 78.1%, and 62.5%, respectively. The average efficiency was 99.1% for each self-healing cycle, indicating the composite hydrogel possessed significant and repeatable electrical restoration performance.

By repeating the complete cutting/self-healing process, without any external force at room temperature, the conductivity of TOCNF-CNT@PANI/PVA-2 hydrogel was tested through *I-V* measurement. Figure 6c shows the time-current flow of TOCNF-CNT@PANI/PVA-2 hydrogel at the same location during repeated cutting/healing processes. In Figure 6d, when the hydrogel was completely cut in half to form an open circuit, the current dropped to zero. Then, the two fractured parts contacted each other, and the current quickly recovered to the initial value through a 20 s in situ self-healing. The conductivity of the hydrogel sample remained stable during the cycle, indicating that the conductivity had a high self-healing efficiency during the cutting-healing process.

As shown in Figure 6e, the self-healing conductive performance of the composite hydrogel was visually displayed through a closed-loop composed of light-emitting diode (LED), TOCNF-CNT/PVA-2 hydrogel, and power components. The LED indicator was lighted with a voltage of 5 V. The LED indicator was extinguished when the TOCNF-CNT/PVA-2 hydrogel was completely separated. However, the LED indicator lit up again, after pushing the two separated parts together for self-healing, illustrating the excellent self-healing conductive property of the composite hydrogel. The hierarchical 3D network consisting of PANI, CNTs, and TOCNFs formed a continuous conducting pathway for electron transport. The dynamically reversible cross-linking points from different borate-induced complexes provided inherent and repeatable self-healing capabilities for hydrogels, exhibiting promise for the self-healing electrode materials [46].

### 3.7. Electrochemical Properties of Composite Hydrogels

**Figure 7:**
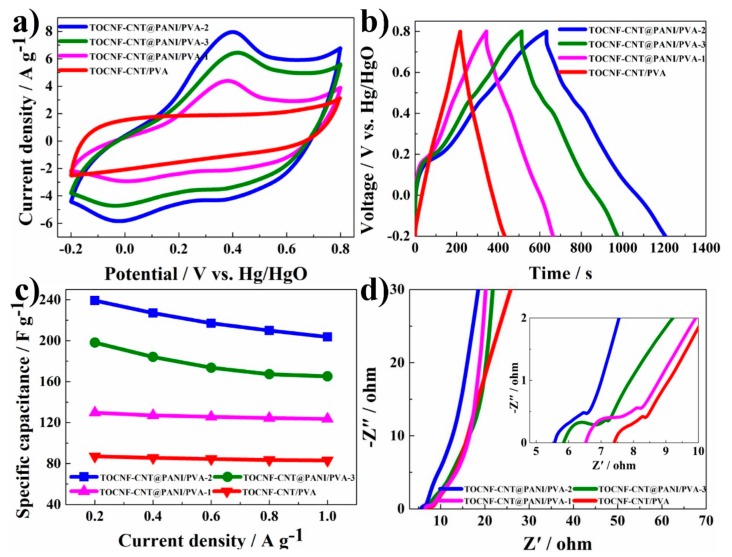
(**a**) CV (cyclic voltammetry) curves at 40 mV s^−1^ scan rate; (**b**) G-CD (galvanostatic charge-discharge) curves at 0.4 A g^−1^ current density; (**c**) specific capacitance of the hydrogel electrodes at different current densities; (**d**) Nyquist plot of hydrogel electrode and enlarged illustration of high-frequency region.

In order to evaluate the effect of the incorporated PANI on the electrochemical behavior of the composite hydrogel electrode, a CV test was performed, as shown in Figure 7a. In the CV test, the potential range was −0.2 to 0.8 V at a scan rate of 40 mV s^−1^, in 6 M KOH electrolyte with platinum sheet counter electrode and Hg/HgO reference electrode. In Figure 7a, the CV curve of TOCNF-CNT/PVA exhibited regular rectangular and symmetric shapes, which reflected the typical characteristics of the electric double layer charge (EDLC) storage. Moreover, the CV curves of composite hydrogel containing PANI possessed a larger current density and different shape. The increase in current density indicated greater capacitance, which was due to the pseudo-capacitance effect of PANI. The deformation of the CV curve was attributed to the diffusion and migration of limited ions in the polymer block and the ohmic resistance due to the thick polymer layer [47]. However, the voltammograms of PANI-based hydrogel possessed clear faradaic oxidation and reduction peaks. Three pairs characteristic peaks arose at 0, 0.4, and 0.6 V; the peaks arose at 0 and 0.6 V were related to the redox behavior of PANI through the leucoemeraldine and pernigraniline states; the peaks at 0.4 V were assigned to the electron transition from the protonation/deprotonation of PANI [14,19]. Among these voltammograms, TOCNF-CNT@PANI/PVA-2 possessed the largest loop area, corresponding to the highest specific capacitance. Furthermore, the G-CD behaviors of these composite hydrogel electrodes were measured at 0.4 A g^−1^ current density from −0.2 to 0.8 V with 6 M KOH electrolyte (Figure 7b). The G-CD curves of the TOCNF-CNT/PVA hydrogel-based electrode exhibited a symmetrical triangle, indicating that it was an electric double-layer capacitor with reversible capacitance characteristics. For all the samples, the G-CD profiles were nearly triangular, demonstrating their excellent capacitive performances. Based on Equation (3), the *C*_s_ was calculated from the G-CD curves data. The *C*_s_ values of TOCNF-CNT/PVA, TOCNF-CNT@PANI/PVA-1, TOCNF-CNT@PANI/PVA-2, and TOCNF-CNT@PANI/PVA-3 were 84.9, 127.3, 226.8, and 184.4 F g^−1^ at 0.4 A g^−1^ current density, respectively. It was observed that composite hydrogel containing PANI possessed a higher specific capacitance than TOCNF-CNT/PVA hydrogel. The 3D network structure of TOCNF-CNT could load PANI and enable greater contact with electrolytes, thereby forming more active sites inside the hydrogel. Moreover, when CNTs were used as the filler for PANI to build a “core-shell” structure composite, the porous structure could further improve the capacitance performance. The high specific capacitance originated from two different charge storage methods: (1) the EDLC storage in CNTs nano-core and (2) the oxidation and reduction chemistry (pseudo-capacitance) of the PANI nano-shell [48].

The specific capacitances of composite hydrogels remained approximately 80%. For all samples, the relationships between the specific capacitance and the current density are shown in Figure 7c. At the same current density, these composite hydrogels containing PANI possessed higher specific capacitances than TOCNF-CNT/PVA hydrogels, indicating that PANI significantly increased the specific capacitance of the composite hydrogel. The sp^2^-hybridized carbon atoms of CNTs formed π-π stacking interactions with the quinoid ring of the PANI without destroying the graphitized plane of CNTs [49,50]. Among these hydrogels, the TOCNF-CNT@PANI/PVA-2 possessed the largest specific capacitance. It could be attributed to the appropriate ratio of PANI to CNTs, which allowed the PANI to better combine with the CNT networks. The developed pore structure and large specific surface area were beneficial to the charge accumulation and enhanced the specific capacitance.

Nyquist plots from EIS of the composite hydrogel electrodes are shown in Figure 7d. The Nyquist plots of hydrogel electrodes showed a typical semicircle in the high-frequency region. The intercept of semicircle represented the equivalent series resistance (ESR), and the diameter of semicircle represented the charge-transfer resistance (*R*_ct_) of the interface. Correspondingly, the diameter of a semicircle of TOCNF-CNT@PANI/PVA-2 hydrogel was the smallest in all samples, indicating that TOCNF-CNT@PANI/PVA-2 hydrogel possessed the lowest resistance. It was because the cross-linked 3D network structure in composite hydrogels provided an ideal charge-transfer path. Nyquist plots showed a straight line at the low-frequency region, and nearly vertical shape reflected the ideal capacitance characteristics [51,52].

### 3.8. Self-Healable and Flexible Performance of the Supercapacitor

**Figure 8:**
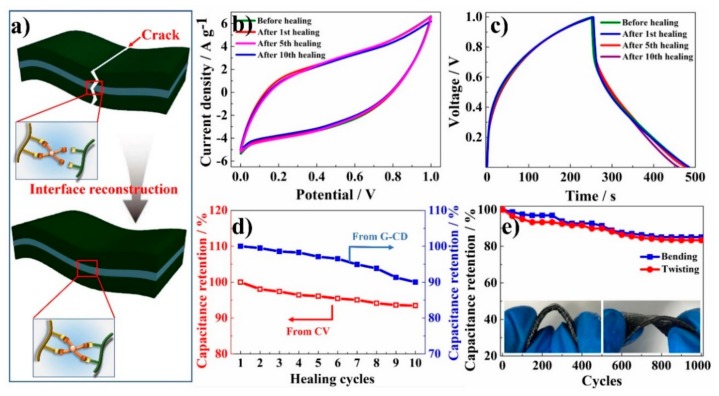
(**a**) Schematic and structural illustration of the self-healing process due to the dynamic borate bond of the supercapacitor. (**b**) CV curves at initial and after the 1st, 5th, and 10th self-healing at a scan rate of 40 mV s^−1^. (**c**) G-CD curves at initial and after the 1st, 5th, and 10th self-healing at a current density of 0.6 A g^−1^. (**d**) Self-healing efficiency derived from both G-CD and CV curves at self-healing cycles from 1st to 10th. (**e**) Capacitance retention over 1000 bending and twisting cycles at an angle of 180°.

The self-healable and flexible solid-state supercapacitor was fabricated based on TOCNF-CNT@PANI/PVA-2 hydrogel electrode and TOCNF/PVA hydrogel electrolyte in a sandwich structure. The detailed fabrication process was described in the experimental part. Due to the inherent flexibility and self-healing ability of the PVA hydrogel, the interfaces between hydrogel electrode and electrolyte could be completely combined, thereby manufacturing an integrated solid supercapacitor device. The assembled supercapacitor could withstand cutting, bending, and another mechanical damage, but the electrochemical performance was not obviously affected. As demonstrated above, the dynamically reversible PVA-borate cross-linking network provided the inherent, repeatable, and effective self-healable ability for the composite hydrogel (Figure 8a).

Figure 8b,c shows the electrochemical performances of the self-healing supercapacitor after multiple cutting/healing cycles. The CV and G-CD curves of supercapacitor had no obvious deformation after multiple cutting/healing cycles, indicating that the capacitance had not been significantly reduced. As calculated by the G-CD curves at 0.6 A g^−1^ current density, the initial specific capacitance of the supercapacitor was 138 F g^−1^, and the specific capacitance after 1, 5, 10 cutting/healing cycles was 137.3, 134.7, and 124.1 F g^−1^, respectively. The corresponding capacitance retention was 99.5%, 97.6%, and 90.0%, respectively. For the as-prepared supercapacitor device, the self-healing capability was more outstanding than those reports. For example, a CNT film was spread on a self-healable substrate to manufacture electrode, combining the self-healable electrodes and polyvinylpyrrolidone-sulfuric acid (PVP-H_2_SO_4_) gel electrolyte to fabricate a supercapacitor. Its capacitance retention reached 85.7% after the 5th cutting/healing cycles [53]. By coating PANI and CNT nanomaterials on the surface of polymer fibers with self-healing ability to develop a novel filamentous self-healable supercapacitor, its capacitance retention was 92% after one cutting/healing cycle [54]. In Figure 8c, the voltage drop in the G-CD curves was due to the electron transfer resistance of the solid-state hydrogel-based electrolyte, which resulted in the specific capacitance of the supercapacitor being smaller than that of the electrode [3].

The in-situ measurement method was used to evaluate the electrochemical performance of the assembled supercapacitor device under the bending or twisting state. Figure 8e shows the variation of capacitance according to the number of cycles, which were calculated from their G-CD curves at a constant current density of 1 A g^−1^ after each bending cycle. A bending cycle started from flat-state, passed through a 180° bending-state, and then returned to flat-state. One twisting cycle was similar. In Figure 8e, the capacitance retention of the supercapacitor device was 85.0% and 82.3% after 1000 bending cycles and twisting cycles, respectively. The performance was comparable supercapacitor to be tested in a flat state. Such a flexible solid-state supercapacitor with PANI hydrogel electrode possessed capacitance retention of 86% after 1000 consecutive charge-discharge cycles [55]. It even was superior to the polyaniline-sodium alginate (PANI-SA) hydrogel supercapacitor reported previously (typically 71% retention for over 1000 cycles) [51]. The improved cycling stability could be due to the quasi-solid hydrogel, further protecting the active PANI and avoiding the delamination of the CNT fibers as conductive pathways. The delamination from continuous expansion and shrinkage of the PANI molecular chain during the charge-discharge cycles could cause performance degradation. The superior capacitance retention under bending/twisting cycles also suggested that the contact between different layers of the supercapacitor device was excellent, which could benefit from the inherent self-healing property of PVA-based hydrogels [43]. The superior flexibility and self-healing solid-state supercapacitor had promising potential applications in a flexible electronic device.

## 4. Conclusions

A unique, flexible, and self-healing ECHs were synthesized through introducing TOCNF-CNT@PANI nanohybrid with a “core-shell” structure into viscoelastic PVA hydrogel matrix. The nanohybrid built a 3D hierarchical framework in the hydrogel matrix, which not only improved the viscoelasticity but also enhanced the electrochemical performance of the ECHs. The ECH possessed a high mechanical toughness (*σ*_s_ ≈ 128 kPa cm^3^ g^−1^ and *E*_e_ ≈ 61 kPa), excellent viscoelastic characteristics (*G*′_∞_ ≈ 18.2 kPa and *G*″_max_ ≈ 7.6 kPa), and ideal electroconductivity (up to 15.3 S m^−1^). The specific capacitance of the TOCNF-CNT@PANI/PVA-2 hydrogel electrode was 226.8 F g^−1^ at a current density of 0.4 A g^−1^. The ECHs exhibited fast self-healing character within 20 s at room temperature and superior flexile performance due to the reversible and dynamic borate-associated network. The symmetric solid-state supercapacitor was fabricated by the TOCNF-CNT@PANI/PVA-2 hydrogel electrodes and TOCNF/PVA electrolyte; the capacitance retention was 90% after 10 cutting/healing cycles; the capacitance retention was 85.0% and 82.3% after 1000 bending and twisting cycles, respectively. Consequently, the novel ECHs provided an alternative platform for personal wearable electronic devices.

## Figures and Tables

**Table 1:** Physical-mechanical characteristics of various hydrogels.

Sample	*σ* at *ε* = 90% [kPa]	*ρ* [g cm^−3^]	*σ*_s_ [kPa cm^3^ g^−1^]	*E*_a_ at *ε* = 90% [kJ m^−3^]	*E*_e_ [kPa]	*W*_c_ [wt%]
TOCNF-CNT/PVA	52.3 ± 0.3	1.14 ± 0.07	~45.9	0.8 ± 0.4	14.4 ± 0.3	95.4 ± 0.2
TOCNF-CNT@PANI/PVA-1	86.1 ± 3.9	1.17 ± 0.12	~73.5	1.7 ± 0.6	38.2 ± 0.6	94.3 ± 0.1
TOCNF-CNT@PANI/PVA-2	152.3 ± 5.1	1.19 ± 0.10	~128.0	3.2 ± 0.5	61.0 ± 0.8	95.15 ± 0.18
TOCNF-CNT@PANI/PVA-3	108.4 ± 4.3	1.23 ± 0.14	~88.1	2.2 ± 0.5	51.7 ± 0.7	94.90 ± 0.14

Note: (1) *σ* means stress; (2) *ε* means strain; (3) *ρ* means density; (4) *σ*_s_ means specific stress; (5) *E*_a_ means energy absorption; (6) *E*_e_ means compressive elastic modulus; (7) *W_c_* means water content.

**Table 2:** Rheological characteristics from viscoelasticity curves.

Parameter	TOCNF-CNT/PVA	TOCNF-CNT@PANI/PVA-1	TOCNF-CNT@PANI/PVA-2	TOCNF-CNT@PANI/PVA-3
*γ*_c_ (%)	2.5	2.1	1.2	1.5
*G*′_max_ (kPa)	2.8	4.0	7.5	5.1
*G*′_∞_ (kPa)	4.3	6.1	18.2	11.8
*G*″_max_ (kPa)	2.6	3.1	7.6	4.5

Note: (1) γ_c_ means critical strain; (2) G′_max_ means the maximum value of storage modulus based on strain; (3) G′_∞_ means the plateau value of storage modulus based on angular frequency; (4) G″_max_ means the maximum value of loss modulus based on angular frequency.

**Table 3:** Tensile strength and tensile rate of hydrogels before and after a 20 s self-healing.

Sample	*F*_original_ (kPa)	*K*_original_ (%)	*F*_healed_ (kPa)	*K*_healed_ (%)	*η*_F (%)_	*η*_K (%)_
TOCNF-CNT/PVA	57.9 ± 2.1	450.5 ± 23.2	56.3 ± 2.0	443.8 ± 22.1	97.2	98.5
TOCNF-CNT@PANI/PVA-1	63.5 ± 2.5	387.8 ± 19.0	61.9 ± 2.4	381.1 ± 18.0	97.5	98.3
TOCNF-CNT@PANI/PVA-2	95.3 ± 3.2	345.1 ± 15.1	92.3 ± 3.7	338.5 ± 13.3	96.8	98.1
TOCNF-CNT@PANI/PVA-3	72.9 ± 2.9	326.7 ± 12.5	69.7 ± 2.8	321.0 ± 10.2	96.7	98.2

Note: (1) *F*_original_ means the break stress of initial hydrogel; (2) *K*_original_ means the break strain of initial hydrogel; (3) *F*_healed_ means the break stress of healed hydrogel; (4) *K*_healed_ means the break strain of healed hydrogel; (5) η_F_ means the healing efficiencies of break stress; (6) *η*_K_ means the healing efficiencies of break strain.
